# Complete Genome Sequence of an H9N2 Influenza Virus Lethal to Chickens

**DOI:** 10.1128/genomeA.00929-14

**Published:** 2014-11-26

**Authors:** Yi Zhang, Xuejing Guo, Juan Qi, Linlin Liu, Jingjing Wang, Shouzhen Xu, Jianlin Wang, Yanbo Yin

**Affiliations:** a College of Animal Science and Veterinary Medicine, Qingdao Agricultural University, Qingdao, China; b China Animal Health and Epidemiology Center, Qingdao, China

## Abstract

An H9N2 virus lethal to chickens was isolated from an acutely ill chicken flock in 2012. Phylogenetic analyses indicated that this virus was phylogenetically related to the Y280 lineage. Sequence analysis showed 1 amino acid deletion in HA1 and 3 amino acid deletions in the NA stalk region.

## GENOME ANNOUNCEMENT

H9N2 influenza viruses have been widely circulating in poultry flocks in Asia and the Middle East, and outbreaks of H9N2 influenza have caused great economic losses (1–4). The H9N2 influenza virus can occasionally transmit to humans, and it was the internal gene donor of the lethal H5N1 influenza virus in 1997 and the H7N9 influenza viruses in 2013 (5–7). Thus, the surveillance of the H9N2 virus was significantly meaningful to the poultry industry and to public health. The H9N2 virus has low pathogenicity to chicken and could not induce obvious signs in specific-pathogen-free (SPF) chickens under laboratory conditions (8, 9). However, coinfection of H9N2 viruses with bacteria could enhance the replication of the H9N2 virus in chickens and cause severe symptoms (10). In an acutely ill chicken flock, we isolated an H9N2 influenza virus and named the strain A/chicken/Shandong/818/2012 (SD/818). In lab experiments, 8 of 10 6-week-old SPF chickens were killed within 10 days, inoculated intravenously with 10^6^ 50% egg infective dose (EID_50_) of the SD/818 virus. However, no chickens that had been infected with the same dose of an earlier isolate, A/chicken/Shandong/1024/2007(H9N2), died.

In order to explore the genome characteristics of the strain, we sequenced the complete genome of SD/818. The results showed that the genome constitutes 8 negative-sense RNA segments: the PB2, PB1, PA, HA, NP, NA, M, and NS genes, with full lengths of 2,341; 2,341; 2,233; 1,739; 1,565; 1,458; 1,027; and 890 nucleotides, respectively. Alignment by BLAST showed that the PB2, PA, HA, NP, NA, M, and NS genes shared the highest nucleotide homologies with H9N2 viruses circulating in Eastern China. However, the PB1 gene of SD/818 shared the highest nucleotide homologies with an H6N8 strain isolated from ducks in Guangxi and an H7N9 strain isolated from a human in Zhejiang. Phylogenetic analyses indicated that the PB1, HA, NA, PA, NP, and NS genes belonged to the Y280 lineage; the M gene belonged to the G1 lineage; and the PB2 gene belonged to an unknown avian lineage.

SD/818 carried the amino acid sequence PSRSSR↓GLF at the HA cleavage site, a character of LPAIV (11). The 226 amino acids (aa) and 228 aa (H3 numbering) of HA receptor-binding sites were Q and L, respectively. The HA carried 8 potential *N*-glycosylation sites at 29 aa, 141 aa, 218 aa, 298 aa, 305 aa, 313 aa, 492 aa, and 551 aa, respectively. Alignment by BLAST showed that the HA protein of SD/818 deleted 1 amino acid at the position of 235, whereas other H9 viruses in GenBank do not show the deletion. The NA showed 3 amino acid deletions at 63 to 65 aa of the stalk region and carried 6 potential *N*-glycosylation sites at 69 aa, 86 aa, 164 aa, 200 aa, 234 aa, and 264 aa. There is a substitution of S31N in the M protein, indicating that this isolate owes its resistance to amantadine (12). The isolate possesses E and D at positions 627 and 701 of the PB2 protein, which is characteristic of viruses of avian origin (13, 14).

In this study, we sequenced the whole genome of an H9N2 virus lethal to chickens and analyzed the characteristic of the genome, which will contribute to exploring the molecular mechanism of the virulence of the H9N2 virus.

### Nucleotide sequence accession numbers.

The genome sequences of A/chicken/Shandong/818/2010 have been deposited in GenBank under the accession numbers KM285394 to KM285401.
